# Age-adjusted charlson comorbidity index score as predictor of prolonged postoperative ileus in patients with colorectal cancer who underwent surgical resection

**DOI:** 10.18632/oncotarget.15285

**Published:** 2017-02-11

**Authors:** Yaohua Tian, Beibei Xu, Guopei Yu, Yan Li, Hui Liu

**Affiliations:** ^1^ Department of Epidemiology and Biostatistics, School of Public Health, Peking University, 100191 Beijing, China; ^2^ Medical Informatics Center, Peking University, 100191 Beijing, China; ^3^ National Healthcare Data Center, Affiliated to National Center for Medical Service Administration, 100191 Beijing, China; ^4^ Hospital Administration Department, Peking University, 100191 Beijing, China

**Keywords:** age-adjusted charlson comorbidity index, prolonged postoperative ileus, colorectal cancer, surgical resection, surgery

## Abstract

Comorbidities had considerable effects on the development of postoperative ileus (POI). The primary aim of the present study was to determine the influence of the age-adjusted Charlson comorbidity index (ACCI) score on the risk of prolonged POI in patients with colorectal cancer who underwent surgical resection. Using the electronic Hospitalization Summary Reports, we identified 11,397 patients with colorectal cancer who underwent surgical resection from 2013 through 2015. Logistic regression models were applied to evaluate the effect of the ACCI score on the risk of prolonged POI. The ACCI score had a positive graded association with the risk of prolonged POI in both colon and rectal cancer (*P* for trend < 0.05). Among patients with rectal cancer, after adjusting for potential confounders, those with an ACCI score of 4–5 had a 108% higher risk of prolonged POI than those with an ACCI score of 0–1 (odds ratio [OR], 2.08; 95% confidence interval [CI], 1.09–3.98), and those with an ACCI score of ≥ 6 had a 130% higher risk (OR, 2.30; 95% CI, 1.08–4.89). Among patients with colon cancer, those with an ACCI score of ≥ 6 had a 47% greater risk of prolonged POI than those with an ACCI score of 0–1 (OR, 1.47; 95% CI, 1.07–2.02). These findings suggested that a higher ACCI score was an independent predictor of the development of prolonged POI.

## INTRODUCTION

Colorectal cancer (CRC) is the third most common cancer and the fourth leading cause of cancer-related death globally. In 2012, an estimated 1.4 million new cases of CRC were diagnosed and 693,900 deaths from CRC occurred worldwide [1, 2]. The incidence of CRC is increasing because of the rapid growth and aging of the population as well as changes in lifestyle factors such as smoking, alcohol drinking, and physical inactivity [3]. An epidemiological study demonstrated that the incidence of CRC was as high as 376.3 per 100,000 and the mortality rate was 191.0 per 100,000 in China in 2015 [4]. Surgical resection remains the primary modality of curative treatment for CRC [5, 6]. Prolonged postoperative ileus (POI) is a problematic and frequent complication following surgical resection of CRC [7]. A recent meta-analysis of 54 studies involving 18,983 patients who underwent colorectal surgery indicated that the incidence of prolonged POI in randomized controlled trials and non-randomized controlled trials was 10.2% and 10.3%, respectively [8]. Furthermore, prolonged POI is closely associated with poorer survival, delayed postoperative recovery, and a prolonged hospital stay and is responsible for considerable patient suffering and substantial health care costs [9–11]. Therefore, preoperative identification of risk factors is important for the prevention of prolonged POI.

Coexisting medical conditions are frequently encountered in elderly patients presenting with CRC [12, 13] and have considerable influences on screening strategies [14], treatment options [15], and postoperative health outcomes [16–18]. Therefore, comorbidities should be identified and adjusted for as confounding factors in clinical outcome studies. A mounting body of evidence has demonstrated independent effects of comorbid conditions including preoperative sepsis, disseminated cancer, peripheral vascular disease, and chronic obstructive pulmonary disease on the incidence of POI [19–21]. However, no studies have used a validated comorbidity score to account for the overall effect of medical comorbid conditions on the risk of prolonged POI after surgical resection of CRC. The Charlson comorbidity index (CCI) is the most widely used method with which to quantify the overall burden of comorbidities and includes 19 medical conditions with corresponding weights [22]. The index has been validated as an effective predictor of health outcomes in patients with CRC who have undergone surgical resection. Higher CCI scores are associated with poorer survival rates, longer length of stay, and increased risk of readmission in patients with CRC after colorectal surgery [23–29]. The age-adjusted Charlson comorbidity index (ACCI) is a modification of the CCI by inclusion of age as one additional comorbidity index. The ACCI has been used for survival prediction and treatment options in patients with CRC [17], bladder cancer [30], and early-stage endometrial cancer [31]. Considering the high incidence of CRC and important clinical significance of prolonged POI and comorbid conditions, it is clinically meaningful to use a validated comorbidity index to quantify the overall effect of comorbid conditions on the risk of prolonged POI in patients with CRC who have undergone surgical resection. Such an index could be used to identify high- or low-risk subgroups for triage for different care trajectories, support individual decision-making, control for the effects of comorbidities in clinical outcomes research, and assess the quality of surgical care.

The primary objective of the present study was to explore for the first time the risk of prolonged POI in patients with CRC who underwent surgical resection with different comorbidity levels as measured by the ACCI.

## RESULTS

Table 1 shows the demographic characteristics of the 5,864 rectal cancer and 5,533 colon cancer patients who underwent surgical resection. The mean age of patients with rectal cancer was 59.66 ± 11.95 years and the mean age of patients with colonic cancer was 60.38 ± 13.30 years. Among patients with rectal cancer, 1,385 (23.6%) patients had an ACCI score of 4–5, and 546 (9.3%) had an ACCI score ≥ 6. Among patients with colonic cancer, 1,509 (27.3%) had an ACCI score of 4–5, and 764 (13.8%) had an ACCI score ≥ 6. Of the 5,864 patients with rectal cancer, 3,118 (53.2%) patients underwent laparoscopic colorectal surgery and 2,746 (46.8%) patients underwent open colorectal surgery; of the 5,533 patients with colon cancer, 2,067 (37.4%) patients underwent laparoscopic colorectal surgery and 3,466 (62.6%) patients underwent open colorectal surgery.

**Table 1:** Demographic characteristics of the 11,397 colorectal patients underwent surgical resection

Variable	Rectal cancer (*N* = 5,864)	Colon cancer (*N* = 5,533)
Age, (year) (mean ± SD)	59.66 ± 11.95	60.38 ± 13.30
Sex		
Men (%)	3,544 (60.4)	3,232 (58.9)
Women (%)	2,320 (39.6)	2,275 (41.1)
Geographic region		
Southern (%)	4,471 (76.2)	4,192 (75.8)
Northern (%)	1,293 (23.8)	1,340 (24.2)
Surgery type		
Open surgery (%)	2,746 (46.8)	3,466 (62.6)
Laparoscopic surgery (%)	3,118 (53.2)	2,067 (37.4)
Anesthesia		
General anesthesia (%)	5,309 (90.9)	5,196 (90.6)
Regional anesthesia (%)	555 (9.1)	337 (9.4)
ACCI		
Mean ± SD	3.06 ± 1.91	3.40 ± 2.27
0–1 (%)	1,103 (18.8)	1,022 (18.5)
2–3 (%)	2,830 (48.3)	2,238 (40.4)
4–5 (%)	1,385 (23.6)	1,509 (27.3)
≥ 6 (%)	546 (9.3)	764 (13.8)

ACCI: Age-adjusted Charlson comorbidity index.

Table 2 presents the prolonged POI occurrences according to ACCI categories. There were 108 (1.8%) and 440 (8.0%) prolonged POI cases in the rectal and colon cancer patients, respectively. In rectal cancer patients with surgical resection, the prevalence rate of prolonged POI increased steadily across ACCI categories, ranging from 1.2% among patients with ACCI score of 0–1 to 1.6% among patients with ACCI score of 2–3, 2.5% among patients with ACCI score of 4–5, and 2.7% among patients with ACCI score ≥ 6. The prevalence rates of prolonged POI in colonic cancer patients with ACCI score of 0–1, 2–3, 4–5 and ≥ 6 were 7.8%, 7.0%, 7.6% and 11.8%, respectively. The Spearman correlation analysis showed that the prolonged POI occurrence was positive related to ACCI groups in both rectal and colon cancer patients who underwent surgical resection (*P* < 0.001).

**Table 2:** Prolonged POI occurrence among 11,397 colorectal cancer patients underwent surgical resection with different ACCI

Variables	Rectal Cancer			Colon Cancer		
	Total	Event (%)	*P* value	Total	Event (%)	*P* value
ACCI	5,864	108 (1.8)	0.029	5,533	440 (8.0)	0.001
0–1	1,103	13 (1.2)		1,022	80 (7.8)	
2–3	2,830	46 (1.6)		2,238	156 (7.0)	
4–5	1,385	34 (2.5)		1,509	114 (7.6)	
≥ 6	5,46	15 (2.7)		7,64	90 (11.8)	

ACCI: Age-adjusted charlson comorbidity index.

Table 3 shows the association between ACCI and the risk of prolonged POI. There was a positive graded association between prolonged POI occurrence and ACCI groups in both rectal (*P* for trend = 0.046) and colonic cancer patients (*P* for trend = 0.002). The risk of prolonged POI increased by an estimated 13.5% (odds ratio [OR], 1.14; 95% confidence interval [CI], 1.04–1.24) and 7.6% (OR, 1.08; 95% CI, 1.04–1.12) for each additional 1 point in the ACCI score in colon and rectal cancer after controlling for sex, geographic region, caseload of each healthcare institution, resection type of surgery, and anesthesia methods, respectively (data was not shown). In rectal cancer with an ACCI score of 4–5 or ≥ 6, there was a 108% and 130% increase in the risk of prolonged POI, respectively, compared with those having an ACCI score of 0–1 (ACCI: 4–5 adjusted OR: 2.08; 95% CI: 1.09–3.98; ACCI ≥ 6: adjusted OR: 2.30; 95% CI: 1.08–4.89), as demonstrated with the use of a logistic regression model. In colonic cancer patients with an ACCI score ≥ 6, there was a 47% increase in the risk compared with those with an ACCI score of 0–1 (adjusted OR: 1.47; 95% CI: 1.07–2.02). Compared with open surgery, laparoscopic surgery was significantly associated lower risk of prolonged POI in both rectal (adjusted OR: 0.68; 95% CI: 0.46–1.00) and colon cancer patients (adjusted OR: 0.48; 95% CI: 0.38–0.60). Regional anesthesia was statistically significantly associated with decreased risk of prolonged POI in patients with colon cancer when compared to general anesthesia (adjusted OR: 0.42; 95% CI: 0.24–0.73).

**Table 3:** Adjusted OR of prolonged POI of colorectal cancer patients underwent surgical resection

	Rectal Cancer			Colon Cancer		
	OR^a^	95% CI	*P* value	OR	95% CI	*P* value
ACCI						
0–1	1			1		
2–3	1.38	0.74–2.56	0.312	0.87	0.65–1.15	0.322
4–5	2.08	1.09–3.98	0.026	0.92	0.68–1.24	0.567
≥ 6	2.30	1.08–4.89	0.030	1.47	1.07–2.02	0.018
*P* value for trend		0.046			0.002	
Geographic Region						
South	1			1		
North	0.97	0.61–1.54	0.909	1.35	1.08–1.69	0.009
Anesthesia						
General anesthesia	1			1		
Regional anesthesia	0.76	0.36–1.62	0.484	0.42	0.24–0.73	0.002
Surgery type						
Open surgery	1			1		
Laparoscopic surgery	0.68	0.46–1.00	0.047	0.48	0.38–0.60	< 0.001
Sex						
Male	1			1		
Female	0.81	0.54–1.22	0.320	0.97	0.80–1.19	0.775

^a^: Adjusted for sex, geographic region, caseload of each healthcare institution, resection type of surgery, and anesthesia methods.

ACCI: Age-adjusted Charlson comorbidity index.

## DISCUSSION

In the present study, age and medical comorbidities as measured by the validated ACCI had a considerable effect on the risk of prolonged POI in patients with CRC who had undergone surgical resection. Patients with a higher ACCI score had a higher risk of prolonged POI. Epidemiologic, quality evaluation, and health services studies aimed at improving the health outcomes of patients with CRC undergoing surgical resection are gaining increasingly more attention [32, 33]. The ACCI promises to be a highly useful tool in such research.

We identified similar studies showing that among patients with CRC, the presence of comorbidities was associated with a higher risk of POI than the absence of comorbidities. Chapuis et al. [19] evaluated 34 potential predictors of prolonged POI in 2,400 patients with CRC who had undergone surgical resection and reported significant impacts of peripheral vascular disease and respiratory comorbidities. Similarly, another study revealed that chronic pulmonary disease contributed to prolonged POI after elective bowel resection in 773 patients with CRC [21]. A recent study involving 27,560 patients who underwent colon resection showed that preoperative sepsis, disseminated cancer, and chronic obstructive pulmonary disease were significantly associated with an increased risk of prolonged POI [20]. In the present study, multivariate logistic regression analysis showed that chronic pulmonary disease and metastatic solid tumors were independent risk factors for prolonged POI. These data suggest that comorbid conditions stimulate the development of prolonged POI. Use of a validated comorbidity index that covers the comorbid conditions frequently encountered in patients with CRC as a tool to quantify the comorbidity burden could provide a more complete assessment of a patient's risk of POI. The present study is the first to propose that the ACCI can be used to help predict the impact of comorbidity on the occurrence of prolonged POI in patients with CRC after surgical resection.

The ACCI may also have value in clinical practice. The ACCI could be used in observational studies as a single index that accounts for age and comorbid conditions; this would eliminate the confounding bias that results from these factors without necessitating the extremely large sample sizes that would be required to control for each risk factor separately [31, 34]. It has been demonstrated that substantial confounding bias would be controlled if strong risk factors could be accurately measured in observational studies [35]. Our study illustrates the need to account for comorbidities. The rates of prolonged POI in the patients with rectal cancer and colon cancer with an ACCI score of ≥ 6 were 125.0% and 51.3% higher, respectively, than those with an ACCI score of 0 to 1. The ACCI may also be helpful in identifying different risk subgroups, leading to more highly tailored approaches to patient management. For example, it could be used to support clinical decision-making for individual patients, such as what treatment option would best suit a specific patient. In addition, using the comorbidity score as a screening tool, patients could be cared for in a suitable setting. Patients with high scores suggesting a significant risk of a complicated resection surgery would receive a high standard of medical care, including both medical equipment and staff. Therefore, the ACCI should be taken into account when planning the individual treatment course. Increasing evidence has demonstrated that individualized rather than standardized post-treatment follow-up care should be performed in cancer survivors [36]. The risk attributable to the overall comorbidity burden may be significant in this context, and the ACCI can be used as a supplementary factor to consider when planning individual surveillance after primary treatment. Studies that evaluate these and other possible clinical applications of the ACCI should be conducted to improve clinical practice.

In the present study, both ICD codes and Chinese terms were applied in the database to check and identify eligible patients and medical conditions. This dramatically reduced the influence of coding inaccuracy, a very important consideration when using administrative health databases [37, 38]. In addition, because all 108 hospitals included in the present study were top-ranked public hospitals, there was limited variation in the hospital setting, hospital quality, surgical strategy, and surgeons’ expertise; this minimized confounding bias.

Our study was subject to certain limitations. First, the retrospective data collection and analysis may bring about corresponding confounding bias. For example, early postoperative small bowel obstruction can be wrongly classified as prolonged POI. On the other hand, some patients with prolonged POI may not be included in the data analysis. However, in China, each patient will receive a comprehensive evaluation of physical and mental health status on postoperative days 3 and 7 and discharge day (the average length of stay for patients with prolonged POI was 21.6 days), therefore, a transient impairment of bowel motility following surgery (< 7 days) or a prolonged POI case was unlikely to be wrongly recorded in our database, minimizing the confounding bias resulting from retrospective data. Additional longitudinal studies, in which more detailed information on prolonged POI cases, and characteristics of comorbidities and colorectal cancer can be prospectively collected, should be conducted to confirm our findings. Second, although the reliability and validity of the ACCI for measurement of comorbidities has been verified, the CCI was initially designed to quantify the overall burden of hospitalized patients and to predict 1-year mortality. Therefore, the ACCI may lack clinically relevant intestinal comorbid conditions, and their weighting algorithms may not necessarily be applicable to patients with CRC who have undergone surgical resection. However, the 19 comorbid conditions included in the CCI cover the overwhelming majority of comorbidities that occur in patients with CRC, and a strong association between the ACCI score and the risk of prolonged POI was observed in the present study. This proves the validity of the ACCI in predicting the risk of prolonged POI. Another limitation was our inability to adjust for the severity of CRC and comorbid conditions because no such information was available in our database. Each of the comorbidities included in the ACCI does indeed have a severity spectrum, and the weight assigned to a specific comorbid condition only reflects the average effect of that condition on the patient's outcome. As a result, such a limitation would potentially affect health outcomes and give rise to residual confounding when it is adjusted for as a confounding factor in research. This also implies that more information on the characteristics of comorbidities should be obtained in clinical application of the index. In some cases, however, calculation of a comorbidity score allowed some uncertainty about the characteristic of comorbid condition [39]. Simplicity and strong operability of an index would be beneficial to ensure routine evaluation of the overall comorbidity burden in the clinical setting, and this was our original aim in the present study. Furthermore, given the robustness of the evidence, statistical analysis, and large sample size in the present study, these limitations are unlikely to have compromised our results.

In conclusion, the risk of prolonged POI increased with a higher ACCI score in patients with CRC who underwent surgical resection. With the increasing proportion of elderly patients and patients with comorbid conditions among those with CRC, consideration of the ACCI could be important in determining the screening strategy and best treatment options as well as preventing prolonged POI among patients with CRC. Future studies should be conducted to test the performance and clinical application of the ACCI in various databases.

## MATERIALS AND METHODS

### Data source

Data used in the present study were extracted from the electronic Hospitalization Summary Reports (HSRs) in the top-ranked public hospitals in care safety and quality as evaluated by the National Hospital Performance Evaluation Project in the National Healthcare Data Center. The standard ranking system considers several aspects including hospital infrastructure, service and management ability, service efficiency, and quality and safety of clinical care. HSRs contain information on basic demographics, admission and discharge dates, hospitalization and discharge diagnoses in Chinese and their corresponding International Classification of Diseases, 10th Revision (ICD-10) codes, surgical operations and their corresponding International Classification of Diseases, 9th Revision, Clinical Modification (ICD-9-CM) codes, outcome of hospitalization (survival status, drug allergy, and hospitalization infection), and financial cost.

An updated HSR was implemented in 2012. The updated HSR contains up to 11 ICD-10 coding fields for discharge diagnoses and up to 10 procedures using ICD-9-CM codes. The first listed diagnosis is available to record the principle diagnosis or primary illness, and other listed diagnoses are available for comorbid conditions and complications. A new variable designed to specify the timing of diagnosis is assigned next to each listed diagnosis. The present study is considered exempt since the data used was collected for administrative purpose without any personal identifiers.

### Study population

In the present study, patients with CRC aged ≥ 18 years were identified from the HSR database from 2013 to 2015 using the ICD-10 codes C18.9 and C20. To minimize the effect of coding inaccuracy, we also used the Chinese terms “rectal cancer” and “colon cancer” to check and identify additional patients with CRC. In total, we identified 64,706 patients with CRC. Next, we identified patients who had undergone surgical resection among these 64,706 patients with CRC using the ICD-9-CM codes 45.7×, 45.8, 45.9×, 48.4×, 48.5, 48.6× and 48.74. Similar to the identification process of patients with CRC, we applied the Chinese terms “resection” and “anastomosis” to identify additional eligible patients. Finally, 11,397 patients with CRC who had undergone surgical resection were included in the present study: 5,864 patients with rectal cancer and 5,533 patients with colon cancer from 108 hospitals in 24 provinces across China.

### Measurements

The health outcome for the present study was prolonged POI. According to Chinese practice [40], prolonged POI is diagnosed based on abdominal plain X-ray and computed tomography, and the presence of clinical manifestations including nausea, vomiting, abdominal pain, abdominal distension, and/or delay in the passage of flatus and stool for more than 7 days postoperatively, in the absence of mechanical bowel obstruction, which is consistent with the definition of prolonged POI used in previous studies [21, 41, 42]. ICD-10 diagnostic code K56 was used to identify prolonged POI cases, and then we used the corresponding Chinese diagnosis to check the identified prolonged POI cases and mechanical bowel obstructions were excluded from this study.

The key independent variable in the present study was the ACCI score. This score was calculated using the algorithm proposed by Charlson et al. [22], in which 19 comorbid conditions were weighted and scored and additional points were added for age (Table 4). The corresponding ICD-10 codes for the 19 comorbidities are listed in Supplementary Table 1. The coding algorithms were carefully checked and reviewed by hospital management experts, physicians, and epidemiologists from our institution and reviewed again by several outside physicians. The ACCI score for each patient was calculated by identifying all comorbid conditions (excluding CRC) and the patient's age and summing the corresponding weights. Higher scores indicate a greater comorbid disease burden. The ICD-10 codes recorded in the HSRs were searched for the 19 comorbid conditions included in the ACCI. Conditions that presented after admission were excluded according to the timing of diagnosis. The age at which surgical resection was performed was adjusted by calculating each decade after 40 years as 1 point in the ACCI. For each decade after 40 years, 1 point was added until 4 points were reached (1 weight for 41–50 years of age, 2 weights for 51–60 years, 3 weights for 61–70 years, and 4 weights for ≥ 71 years).

**Table 4:** Age-adjusted charlson comorbidity index

Weight	Comorbid Condition
1	Myocardial infarction
	Congestive heart failure
	Peripheral vascular disease
	Cerebrovascular disease
	Dementia
	Chronic pulmonary disease
	Connective tissue disease
	Ulcer disease
	Mild liver disease
	Diabetes
2	Hemiplegia
	Moderate or severe renal disease
	Diabetes with end organ damage
	Any solid tumor
	Leukemia
	Lymphoma
3	Moderate or severe liver disease
6	Metastatic solid tumor
	Acquired immunodeficiency syndrome (AIDS)
1	For each decade over age 40 years, up to 4

### Statistical analysis

In the present study, patients were categorized into four groups based on their ACCI score: 0–1, 2–3, 4–5, and ≥ 6 [17]. Categorical variables are reported as proportion (%), and numerical data are reported as mean ± standard deviation. Differences were analyzed with the t test for numerical variables and Pearson's χ^2^ test for categorical variables. The incidences of prolonged POI between different ACCI groups were compared using Pearson's χ^2^ test. The Cochran–Armitage test for trend was used to analyze the association between the occurrence of prolonged POI and the ACCI score. We used prolonged POI as a dependent variable and a series of multivariable logistic regression models to calculate the odds ratios (ORs) and 95% confidence intervals (95% CIs) across all ACCI groups. Variables potentially related to POI formation that were considered in the model were sex, resection type of surgery (laparoscopic vs. open resection), geographic region (south vs. north), caseload of each healthcare institution and anesthesia methods (general vs. regional anesthesia). All reported *P* values were nominal and two-sided. All statistical analyses were performed by R software.

## SUPPLEMENTARY MATERIALS TABLES


