# Supplementary material for: Age-adjusted charlson comorbidity index score as predictor of prolonged postoperative ileus in patients with colorectal cancer who underwent surgical resection
Source: Oncotarget. 2017 Feb 11;8(13):20794–801. doi: 10.18632/oncotarget.15285 (PMC5400545; doi:10.18632/oncotarget.15285)
Supplement: Supplementary file 1 [file oncotarget-08-20794-s001.pdf]

Age-adjusted charlson comorbidity index score as predictor of prolonged postoperative ileus in patients with colorectal cancer who underwent surgical resection

Supplementary Materials

Supplementary Table 1: ICD-10 coding algorithms for age-adjusted charlson comorbidity index

Comorbidity	ICD-10
Myocardial infarction	I21, I22, I23, I24, I25
Congestive heart failure	I09.9, I11.0, I13.0, I13.2, I42, I43, I50, I51.7, I26
Peripheral vascular disease	I70-I74, I77, I79.0, I79.2, I80, K55.1, K55.8, K55.9, Z95.1, Z95.5, Z95.8, Z95.9,
Cerebrovascular disease	G45, G46, H34.0, I60- I69
Dementia	F00-F03, F05.1, G30, G31.0, G31.1
Chronic pulmonary disease	I27.8, I27.9, J40-J47, J60-J67, J68.4, J70.1, J70.3, J84, J94, J96, J98
Connective tissue disease	L94.0, L94.1, L94.3, M05, M06, M08, M12.0, M12.3, M30-M36, M45, M46.1, M46.8, M46.9
Ulcer disease	K25-K28, K92
Mild liver disease	B18, K70.0-K70.3, K70.9, K71.3-K71.5, K71.7, K71.8, K73-K76, R94.5
Diabetes	E10.0, E10.1, E10.5, E10.6, E10.8, E10.9, E11.0, E11.1, E11.5, E11.6, E11.8, E11.9, E12.0, E12.1, E12.6, E12.8, E12.9, E13.0, E13.1, E13.5, E13.6, E13.8, E13.9, E14.0, E14.1, E14.5, E14.6, E14.8, E14.9
Hemiplegia	G04.1, G11.4, G80.1, G80.2, G81-G83
Moderate or severe renal disease	I12, I13.1, I13.9, I15, N00, N01, N03, N05, N07.2-N07.4, N13.3, N17-N20, N25, N28, N39, N40, Z49.0-Z49.2, Z94.0, Z99.2
Diabetes with end organ damage	E10.2-E10.4, E11.2-E11.4, E13.2-E13.4, E14.2-E14.4
Any solid tumor, leukemia and lymphoma, except colorectal cancer	C00-C14, C30-C34, C37-C41, C43, C45-C58, C60-C76, C80-C86, C88, C90-C97, Z85
Moderate or severe liver disease	B15.0, B16.0, B16.2, B19.0, I85, I86.4, I98.2, K70.4, K71.1, K72
Metastatic solid tumor	C77-C79
AIDS	B20-B24

AIDS: Acquired immunodeficiency syndrome.
